# The Significant Influence of Bacterial Reaction on Physico-Chemical Property Changes of Biodegradable Natural and Synthetic Polymers Using *Escherichia coli*

**DOI:** 10.3390/polym9040121

**Published:** 2017-03-25

**Authors:** Chankyu Kang, Sam Soo Kim, Soo Jung Kim, Jaewoong Lee

**Affiliations:** 1 Ministry of Employment and Labor, Major Industrial Accident Prevention Center, 34 Yeosusandallo, Yeosu-Si, Jeonnam 59631, Republic of Korea; chemnet75@korea.kr; 2 Department of Textile Engineering and Technology, Yeungnam University, 280 Daehak-Ro, Gyeongsan, Gyeongbuk 38541, Republic of Korea; sskim@yu.ac.kr (S.S.K.); sjkim@yu.ac.kr (S.J.K.)

**Keywords:** bacterial hydrolysis, biodegradation, tensile strength, contact angle, XPS, physico-chemical property

## Abstract

*Escherichia coli* (*E. coli*) was used to activate hydrolysis reaction along with biodegradation in natural and synthetic fibers to identify possibilities as alternative substitutes for textile wastes using chemical solutions and enzymes. To confirm the reaction between the bacterial infections of *E. coli* and the excessively abundant interstitial spaces of the fibers, various types of natural and synthetic fibers such as cotton, wool, polyethylene terephalate (PET), polyadmide (PA), polyethylene (PE), and polypropylene (PP) were used to confirm the physico-chemical reactions. Tensile strength analysis, scanning electron microscopy (SEM), X-ray photoelectron spectroscopy (XPS), and contact angle analysis were used to determine the physico-chemical property changes of the fiber by the bacteria. When biofilm was formed on the fiber surface, various physical changes such as the following were observed: (i) in the analysis of tensile strength, all except PA and PP were decreased and a decrease in cotton fibers was noticeable (ii) depending on the type of fibers, the degree of roughness was different, but generally the surface became rough. In this study, the change of roughness was the most severe on the cotton fiber surface and the change of PET and PA fiber was relatively small. It was found that the intensity peak of oxygen was increased, except for the in cases of PA and PP, through the change of chemical properties by XPS analysis. Changes in topographical properties on the surface through contact angle analysis were stronger in hydrophilic properties, and in the case of cotton, completely hydrophilic surfaces were formed. Through this study, PA and PP fibers, which are Olefin fibers, were theoretically free of physicochemical and topographical changes since there were no functional groups that could trigger the hydrolysis reaction.

## 1. Introduction

Textile materials, especially those used in hospitals, infant wear, and underwear, have become a great health concern because they have direct contact with the human body [1]. Microbial organisms such as bacteria multiply rapidly and cause territorial expansion of textile materials in a particular environment [2,3]. Sweat in the human body and high humidity in the environment promote the growth of microorganism [4]. The generation of odor, loss of performance, or discoloration of textile materials are the results of microbial infestation [5,6,7]. The first step of all those problems begins with bacterial adherence on fiber surfaces and leads to further growth, proliferation, biofilm formation, and degradation of fiber materials [8].

Textile processing with biotechnology using microorganism such as enzymes has been recognized as an alternative technology for reducing hazardous chemicals. Because the traditional chemical treatments result in problematical low product quality, higher manufacturing cost, harmful waste, and enormous energy consumption, technologies using eco-friendly enzymes provide industrial safety along with completely biodegradable without environmental pollutants [9,10,11,12,13]. Several types of enzymes such as amylases, cellulases, lipases, proteases, esterase, nitrilases, catalases, peroxidases, laccases, and pectin-degrading enzymes have been used in textile processing (i.e., desizing, bio-ploishing) [14]. Because the application of enzymes requires a high concentration of enzymes and long hydrolysis times, many studies have been focused on compensating for suggested disadvantages [15,16]. 

Hydrolysis reaction has been induced by using enzymes in natural and synthetic polymeric fibers, for example cotton [17,18], wool [19], polyethylene terephthalate (PET) [20,21], and polyamide (Nylon) [22]. The reason why the hydrolysis reaction is interesting is associated with transition of physical and chemical properties as well as biodegradation. For example, hydrogen bonding was gradually increased in enzymatic hydrolysis using crude cellulases from *Trichoderma pseudokoningii* S-38 in cotton fibers [23]. Significant structural changes appeared on the cotton surface formed long and deep holes on the fiber surface which induced a rougher surface as extended time of enzymatic hydrolysis. In the study of enzymatic and alkaline hydrolysis of PET fibers, the change of surface property was detected and enzyme played the role of catalyst [24]. The increase of the reaction rate by enzyme can be very useful in the bio-polishing process to make soft and the color bright textile fabrics.

*E. coli* is a gram-negative bacterium that is a major cause of bacterial infections. Most of them are not harmful, but some strains have adverse effects on human health such as anemia, cholecystitis, cholangitis, diarrhea and urinary tract infections [25]. Because of the abundant interstitial space between fibers, the growth and adhesion behavior of *E. coli* on the fiber surface has been a major challenge to human health. In order to solve this problem, several studies have been carried out to inhibit the release of active species using quaternary ammonium, chitosan, silver and TiO_2_ that react with the surface [26,27,28]. 

The main purpose of this study was biodegradation of fiber polymer by *E. coli* and its secretion. In addition, this study aimed also to identify the feasibility of using *Escherichia coli* to alternative enzymes and chemical solutions used mainly in textile waste treatment of natural and synthetic fibers. *E. coli* has made full use of the fact that it spreads easily among people and thus also affects the physico-chemical properties of the fibers. To the best of our knowledge, this study was the first application of *E. coli* to fiber polymer degradation. One of the most studied bacteria among the many species of bacteria is *E. coli*. There has been no study of the effect of *E. coli* and its secretion on the polymer, especially on the fiber polymer. Several types of natural and synthetic fibers were used and their physico-chemical properties along with biodegradation phenomenon were investigated. The bacteria, *Escherichia coli* (*E. coli*), were directly deposited and grew on the cotton, wool, polyethylene terephthalate (PET), polyamide (Nylon), polyethylene (PE), and polypropylene (PP). Surface morphology, physico-chemical properties and chemical composition were determined by scanning electron microscopy (SEM), tensile strength, X-ray photoelectron spectroscopy (XPS), and contact angle measurement. 

## 2. Materials and Methods

### 2.1. Materials

Naïve cotton, wool, PET, Polyamide (Nylon), and Polypropylene (PP) fabrics were 115 g/m^2^ (ISO 105-F02), 125 g/m^2^ (ISO 105-F01), 130 g/m^2^ (ISO 105-F04), 130 g/m^2^ (ISO 105-F03), and 170 g/m^2^ (Style No. 983), respectively, and were purchased from Testfabrics Korea (Ansan, Korea). Polyethylene (PE) fabric (290 g/m^2^) was provided by the Sinpung Textile (Daegu, Korea). All chemicals used in this study were purchased from Duksan Chemicals (Ansan, Korea) and used without additional further purifications.

### 2.2. Sample Preparation

The process of forming a biofilm on a given sample was composed of several steps. To prepare the control samples, the samples were pre-cleaned several times with ethanol solution followed by rinsed with water (80 °C) to remove finishing agents and then dried at room temperature for 24 h. The test samples used in the experiment were also used after the same pre-cleaning processes as the control sample. After these processes, *Escherichia coli* (*E. coli*) (KCCM 12451), grown into Luria-Bertani agar (BD Biosciences, Franklin Lakes, NJ, USA) broth for 37 °C at 18 h, was inoculated into fibers. The inoculation times of the samples were varied and the change of the surface roughness was observed by SEM. Before XPS and contact angle measurement, the samples were dipped into 75% ethanol solution for 10 min and then soaked in 1% sodium hypochlorite solution for 30 min. These samples were rinsed in distilled water and dried at room temperature to remove any remaining residuals. 

### 2.3. SEM Analysis

The influence of bacteria hydrolysis on the surface morphology was examined by SEM (S-4100, Hitachi Co. Ltd., Tokyo, Japan) after the inoculation of bacteria were formed in their colonies for 5 days. The samples treated with bacteria were soaked in formaldehyde solution (4%) for 30 min to immobilize the morphology of bacteria followed by SEM measuring. To obtain a better image, the samples were pre-coated with gold by sputtering on the surface and SEM was operated at 15 kV with 4000× magnification.

### 2.4. Mechanical Strength 

The physical strength of the fibers was monitored after biofilm was formed. During the measurement of tensile strength, the experiment was carried out quickly with the sample prepared to prevent the formation of additional biofilm. The tensile strength was measured by universal testing machine (Model 3345, Instron Corp., Canton, MA, USA) with a 100 N load cell and pneumatic-action grips No. 2712-002. The distance between two grips was 50mm and at least five measurements were repeated for each sample. 

### 2.5. XPS Analysis

The binding energy of the elemental components was analyzed by XPS using a Quantera SXM (ULVAC-PHI, Ulvac, Tokyo, Japan) for determining the change of chemical composition by bacteria.

### 2.6. Surface Property

The change in the characteristics of the surface profile was measured using Dataphysics OCA 20 (Dataphysics, Regensburg, Germany) instrument at room temperature. The comparison of the sample with/without bacteria on the sample surface was performed on using the sessile drop method (temperature: 19–22 °C, relative humidity: 40%). In this process, 2 µL of deionized water was dropped on the target surface and the contact angle was measured. The process was repeated 10 times to reduce the experimental error (± 2°).

## 3. Results and Discussion

### 3.1. Analysis of Physical Property

**Figure 1:**
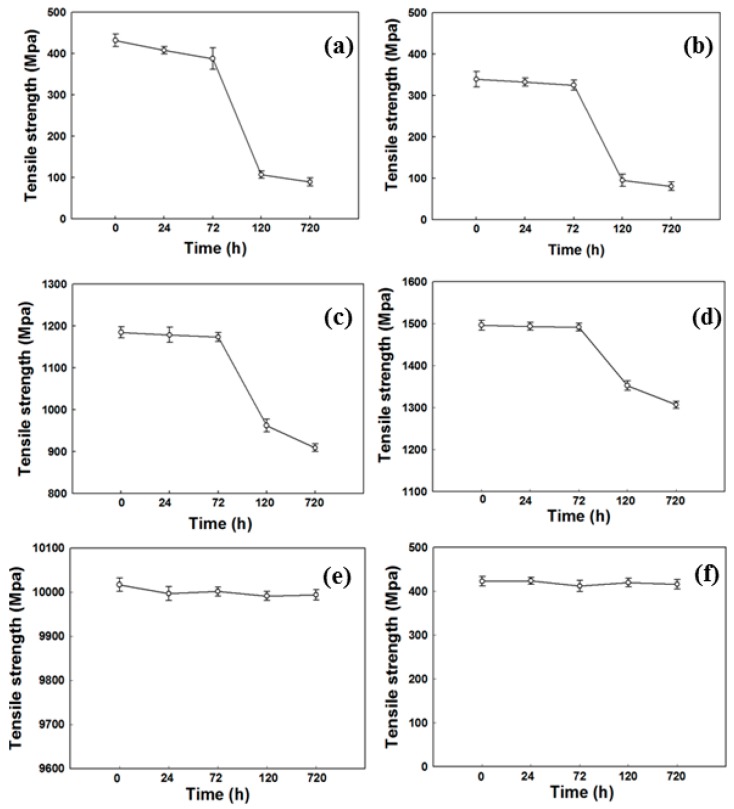
Tensile strength analysis of fiber by reaction with *E. coli* (**a**) cotton; (**b**) wool; (**c**) polyethylene terephalate (PET); (**d**) polyadmide (PA); (**e**) polyethylene (PE) and (**f**) polypropylene (PP) fibers.

The tensile strength test was used as a touchstone to determine the physical properties of various fibers by bacteria and the results were presented in Figure 1. The change began at the third day of tensile strength and maximum reduction was detected at fifth day except for PE and PP. The largest tensile strength in this study was cotton, one of the natural fibers, and the change in synthetic fibers was relatively small except for PET. This means that bacteria react with fiber materials after a certain period of time after the bacteria form the biofilm. These results indicate that *E. coli* plays a significant role in weakening the tensile strength. Similar results have been reported when using chemical solutions or enzymes that are used in fibers [29,30,31]. Ji et al. [29] reported that the tensile strength was weakened by cross-linking the case with formaldehyde. In addition, studies using enzymes dissolved and destructed materials similar to those used with chemicals [30,31]. A less significant influence was found in PA, a type of synthetic polymers. In the case of PE and PP fibers, the change of tensile strength was negligible over time. The reason for this phenomenon was that olefin polymers such as PE and PP theoretically did not have functional groups which could theoretically cause hydrolysis reaction, and therefore there was no physical property changes even after biofilm was formed, as shown in Figure 1e,f. It is known that the moisture in cotton and wool allows the bacteria to easily penetrate amorphous regions and leads to active hydrolysis reaction [32]. Therefore, it can be concluded that *E. coli* reacts with the functional groups on the fiber surface and weakens the tensile strength. This decrease in tensile strength is closely related to biodegradation phenomenon and improves biodegradability [33].

### 3.2. Surface Morphology 

**Figure 2:**
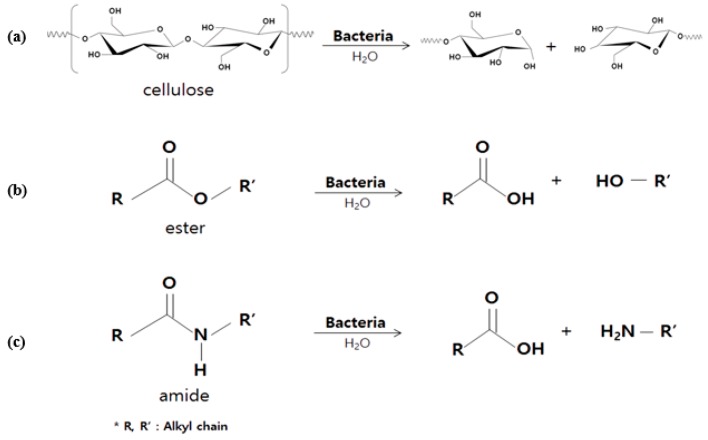
Schematic diagrams of bacterial hydrolysis for (**a**) cellulose; (**b**) ester and (**c**) amide functional groups.

**Figure 3:**
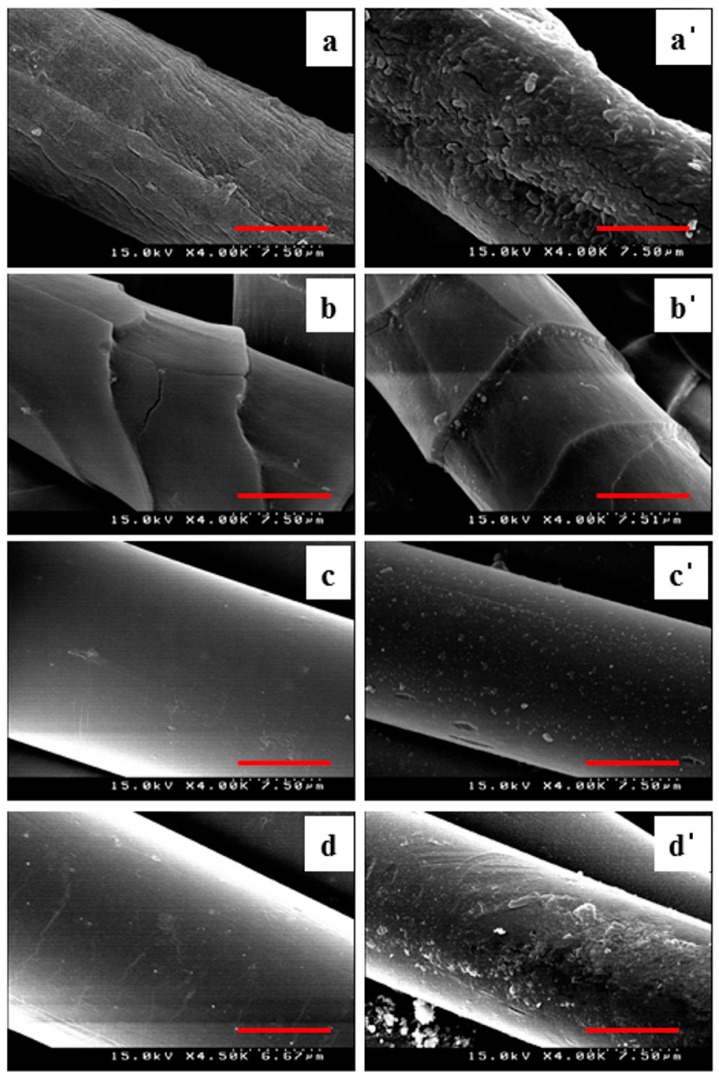
Scanning electron micrograph of native fibers; (**a**) cotton; (**a’**) cotton treated with bacteria; (**b**) wool; (**b’**) wool treated with bacteria; (**c**) PET; (**c’**) PET treated with bacteria and (**d**) PA and (**d’**) PA treated with bacteria (The length of the scale bar used here was 7.5 µm).

The reaction of cellulose, ester, and amide functional groups using *E. coli* was suggested in Figure 2 as a schematic diagram. These reactions contributed to the physico-chemical property change of the fiber materials. Surface morphology changes of natural and synthetic fibers through SEM image analysis clearly showed the effect of bacteria, as shown in Figure 3. 

Regardless of the material of the biofilm fibers, except for PE and PP fibers, it was confirmed that biofilm- formed fibers exhibited relatively rough surfaces compared to the control samples, and the degree of natural fibers was much higher than that of synthetic fibers. This means that the reactivity of the bacteria strongly depends on the fiber materials. For example, many holes and cracks were observed in the case of cotton fiber, as shown in Figure 3a, whereas in the case of PET a smooth surface was observed in Figure 3c. Cotton would have been a lot of reactions by bacteria because it has a good structure to be induced strong bacterial adhesions [5]. Therefore, the creation of a rough surface was the result of increased reactivity on the surface due to the properties of the fiber itself. Figure 3b showed the analysis of the wool fiber. The effect of the surface roughness by the bacteria actually existed but was very imperceptible. This is probably similar to the result of using an enzyme on the wool surface. Bahi et al. [34] reported that the wool treatment with enzyme observed in a very slight partial damage to the wool surface, resulting in non-uniform surface roughness. As a result of this study, it seems that *E. coli* has also performed a similar role to account for imperceptible damage on the wool. SEM analysis also showed that increasing the etched wrinkle on the surface was detected in the PET sample, as shown in Figure 3c. This phenomenon was similar to the alkaline hydrolysis reaction induced by NaOH, resulting in a softer, partially damaged surface [35]. As a result, *E. coli* also played a role similar to NaOH and reacted with the polymer chain of PET to produce etched marks on the surface. Since the bacteria on the surface of PA fabrics was not uniformly distributed but rather located at a specific location, it was assumed that the observed surface appeared to be rough in Figure 3d. This phenomenon is caused by the behavior of bacteria to form a cluster. Through this study, surface property analysis using SEM images revealed that *E. coli* reacted with several fibers to form a rough surface.

### 3.3. Reaction of E. coli with Fiber Materials 

**Figure 4:**
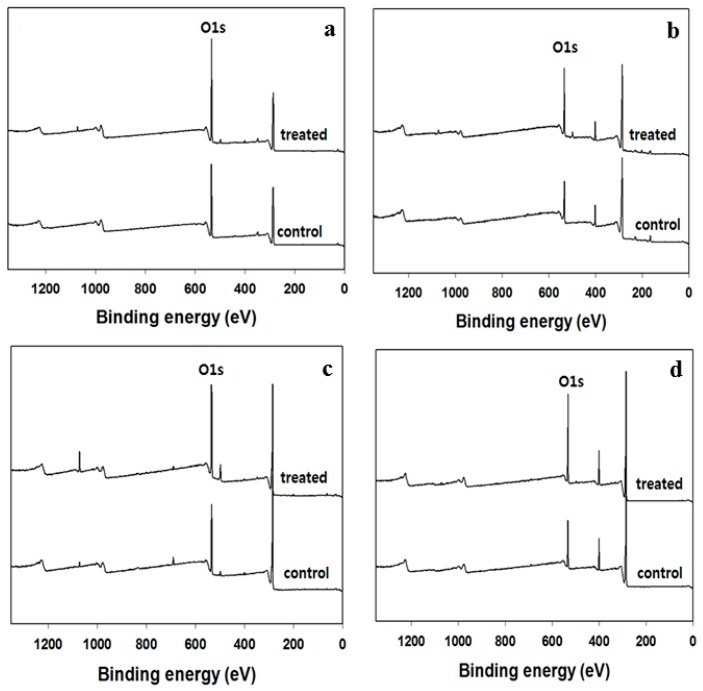
X-ray photoelectron spectroscopy (XPS) analysis of (**a**) cotton; (**b**) wool; (**c**) PET and (**d**) PA.

The reaction by *E. coli* after 5 days of treatment was initially determined by FT-IR presented in the supplemental data (Figure S1). Ether, ester, and amide groups contained in cotton, wool, PET, and PA decreased while hydrolysis or amide groups were found to increase through FT-IR analysis. By inoculation of the bacteria, the -OH group near 3200–3600 nm was changed, and it was confirmed that hydrolysis occurred by the analysis using FT-IR. Unfortunately, this analysis did not provide meaningful data, but rather indicated probability, so XPS analysis was performed to confirm the increase of oxygen groups on the surface of the sample. The peak spectrum analysis of the control sample and *E. coli* treated sample, as shown in Figure 4, revealed that the intensity of the oxygen peak was increased in the case of the *E. coli* treated sample. It was known that 531 eV in XPS spectrum indicated the presence of an oxygen peak [36]. The intensity of the oxygen peaks for cotton, wool, PET, and PA samples was increased after bacterial treatment followed by leaving for 720 h at ambient temperature. Cross-linking reaction and acid degradation of cellulose were observed simultaneously when acid solutions were used. As a result, the increase of oxygen peak and the decrease of strength were found [36]. In this study, we conclude that *E. coli* plays a similar role to acid solutions because the oxygen peak intensity and tensile strength decrease. 

### 3.4. Topographical Properties of the Surface 

**Figure 5:**
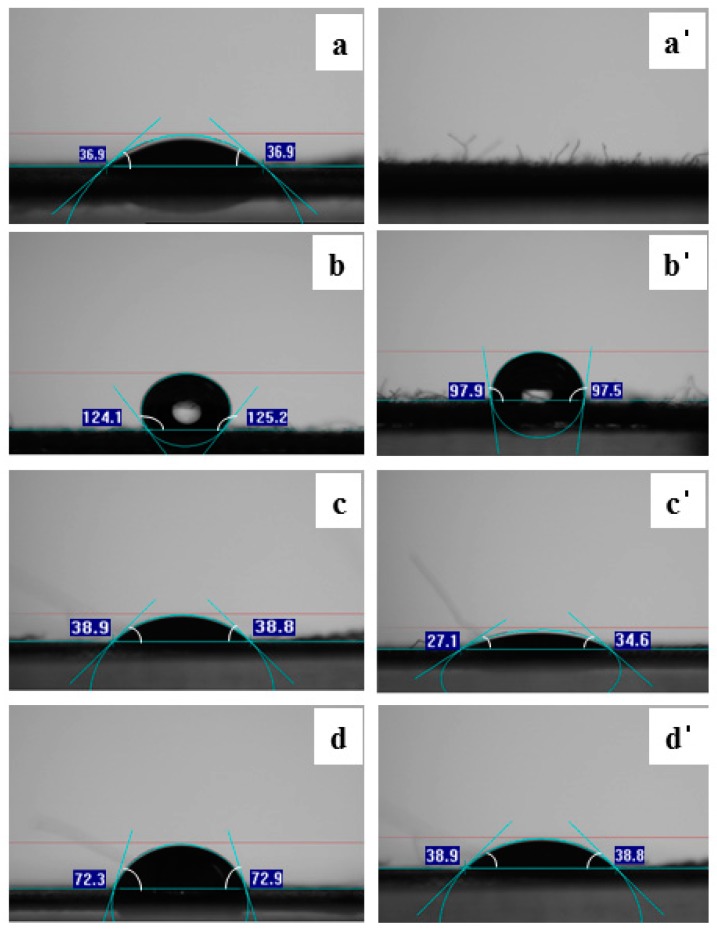
Contact angle measurement of (**a**) cotton; (**a’**) cotton treated with bacteria for 5 days; (**b**) wool; (**b’**) wool treated with bacteria for 5 days; (**c**) PET; (**c’**) PET treated with bacteria for 5 days and (**d**) PA and (**d’**) PA treated with bacteria for 5 days.

The change of the hydrophilicity of the surface by *E. coli* was suggested by analysis of the contact angle. It was well known that contact angle measurement after hydrolysis reaction modified the property of hydrophobicity [37]. Figure 5a showed the change in the contact angle measurement of the cotton. The contact angle before treatment of *E. coli* was 36.9, and after treatment the contact angle was not measured because the surface was completely hydrophilic. The reduction of the smallest contact angle was observed in PET fibers. Contact angle measurements in this study were different for all types of fibers but decreased for all fibers except for PE and PP fibers used in the experiment. This phenomenon is associated with the formation of amine and hydroxyl groups as *E. coli* scissoring the molecular chains of the fibers. Therefore, the hydrophilic property on the surface increased. Although PET and PA Fibers have relatively low contact angles due to the high crystallinity of the fiber itself compared to natural fibers, the reaction between *E. coli* and amide and ester groups reduces the contact angle at the surface. 

## 4. Conclusions

*E. coli*, which is the most common causative organism and a highly contagious bacterium among humans, has changed the physico-chemical properties of fibers along with biodegradation by causing reactions similar to chemical solutions and enzymes. SEM images and XPS analysis provides evidence that bacteria, existing in fibers, induce a rough surface and promote a hydrolysis phenomenon. Through SEM images and XPS analyses, *E. coli* were induced to increase the reactivity at the fiber surface, roughening the surface and increasing the peak intensity of oxygen. The surface morphology of biofilm-formed fibers by SEM analysis confirmed that many holes and cracks were observed in the case of cotton fiber because it had a good structure to be induced strong bacterial adhesions and caused surface reaction. Tensile strength was also strongly dependent on types of materials and bacteria exposure times. Significant reductions in tensile strength were detected 5 days after biofilm formation, which means that the reaction takes a certain amount of time to proceed by *E. coli*. The greatest reduction of tensile strength was found in cotton, while the decrease in wool, a natural fiber, was relatively small. Overall, the decrease in tensile strength in synthetic fibers was not significant except for PET fibers, and no significant reduction was observed in PE and PP fibers, as there were no functional groups that could react with *E. coli* in Olefin fibers. As a result, *E. coli* and its secretion can be applied to the decomposition of condensation polymers that have not previously been studied and can be used to effectively degrade the polymer. In addition, the addition polymer has been proved to have little influence. The XPS analysis showed that the peak intensity of the oxygen group on the sample surface increased. It seems that moisture contents of fibers were a strong factor to give rise to induce hydrolysis reaction. While many changes in the contact angle were detected in the cotton, relatively little change was detected in the PET fiber. From this study, *E. coli* plays a role similar to chemical solutions and enzymes, causing a hydrolysis reaction and biodegradation, and results in different outcomes depending on the fiber material, the adherence of the bacteria, and the exposure time. 

## Supplementary Materials

The following is available online at www.mdpi.com/2073-4360/9/4/121/s1. Figure S1. FT-IR analysis result (**a**) cotton; (**b**) wool; (**c**) PET and (**d**) PA.
