# Supplementary material for: The Significant Influence of Bacterial Reaction on Physico-Chemical Property Changes of Biodegradable Natural and Synthetic Polymers Using Escherichia coli
Source: Polymers (Basel). 2017 Mar 25;9(4):121. doi: 10.3390/polym9040121 (PMC6431910; doi:10.3390/polym9040121)
Supplement: Supplementary file 1 [file polymers-09-00121-s001.pdf]

The Significant Influence of Bacterial Reaction on Physico-Chemical Property Changes of Biodegradable Natural and Synthetic Polymers Using *Escherichia coli*

Chankyu Kang, Sam Soo Kim, Soo Jung Kim and Jaewoong Lee

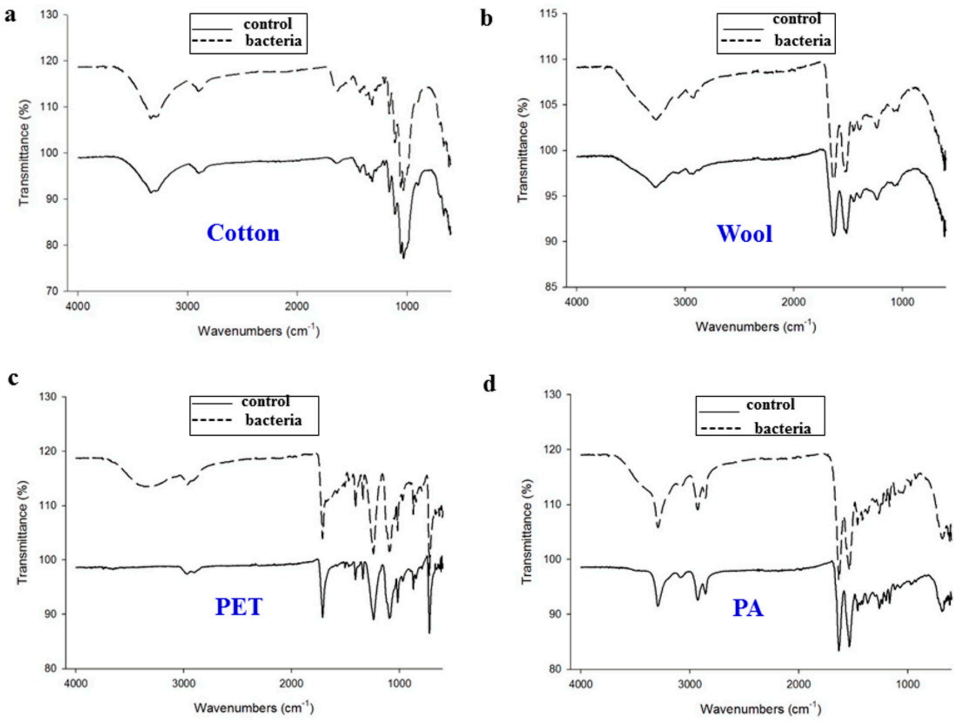

Figure S1. FT-IR analysis result (**a**) cotton; (**b**) wool; (**c**) PET and (**d**) PA.
